# A novel local field potential-based functional approach for targeting the centromedian-parafascicular complex for deep brain stimulation

**DOI:** 10.1016/j.nicl.2021.102644

**Published:** 2021-03-26

**Authors:** Jackson N. Cagle, Robert S. Eisinger, Marshall T. Holland, Kelly D. Foote, Michael S. Okun, Aysegul Gunduz

**Affiliations:** a J. Crayton Pruitt Department of Biomedical Engineering, University of Florida, Gainesville FL, United States; b Department of Neuroscience, University of Florida, Gainesville, FL, United States; c Department of Neurosurgery, University of Florida Norman Fixel Institute for Neurological Diseases, Gainesville, FL, United States; d Department of Neurosurgery, University of Iowa, Iowa City, IA, United States; e Department of Neurology, University of Florida Norman Fixel Institute for Neurological Diseases, Gainesville, FL, United States

**Keywords:** Centromedian nucleus, Deep brain stimulation, Tourette syndrome, Stereotactic targeting, Local field potential, Event-related potential

## Abstract

**Background:**

The centromedian-parafascicular (Cm-Pf) complex of the thalamus is a common deep brain stimulation (DBS) target for treatment of Tourette syndrome (TS). Currently, there are no standardized functional intraoperative neurosurgical targeting approaches. Collectively, these issues have led to variability in DBS lead placement. Therefore, more defined methods are needed to improve targeting accuracy.

**Objective:**

The objective of this observational study was to develop and to verify a functional mapping task capable of differentiating the Cm-Pf region from the nearby ventral intermediate (Vim) nucleus region of the thalamus. The overarching goal was to improve the reproducibility of DBS targeting in the Cm-Pf region.

**Methods:**

Seven TS patients completed a modified Go/NoGo task (five in the post-operative setting and two in the intra-operative setting). Post-operative neural signals from Cm-Pf region were collected using sensing-enabled implanted neural stimulators, and intraoperative neural signals from the Cm-Pf region were collected using an external amplifier. Event-related potential (ERP) features were identified by using the grand-average of stimulus onset signals derived from the postoperative participants. These features were correlated with anatomical locations for the specific electrode recordings. The same features were extracted from the intraoperative patients in order to verify electrode positions in the operating room environment.

**Results:**

Two features – a positive and a negative deflection – were identified in the average ERP from the post-operative participants. The peak amplitudes of both features were significantly correlated with the electrode depth position (p = 0.025 for positive deflection and p = 0.039 for negative deflection). The same result was reproduced intra-operatively in the two most recent patients, where more ventral electrode contacts revealed stronger peak amplitudes in comparison to the dorsal electrode contacts.

**Conclusion:**

This process was used to physiologically confirm accurate lead placement in the operating room setting. The modified Go/NoGo task elicited robust neural responses in the Cm-Pf region however the signal was not present in the Vim nucleus region of thalamus along the DBS electrode trajectory. We conclude that the differences in ERP responses may be a potentially novel LFP based functional approach for future targeting of the Cm-Pf complex for TS DBS.

## Highlights

• A functional targeting method for centromedian-parafascicular nucleus of thalamus.
• Strong local field potentials that correlate with position during stimuli onset.
• Differentiable signal along the trajectory of the electrode in thalamus.

## 1 Introduction

Deep brain stimulation (DBS) is a surgical procedure commonly used to treat movement disorders such as Parkinson’s disease, dystonia and essential tremor (Eisinger et al., 2019). Recently, DBS indications have expanded to include neuropsychiatric disorders such as Tourette syndrome (TS) (Goodman and Alterman, 2012). TS is a neurodevelopmental disorder characterized by involuntary motor and vocal tics. Although for most patients, the tic symptoms subside by adulthood, some patients experience persistent and medically-refractory symptoms (Wand et al., 1993). Based on the basal ganglia-thalamocortical loop dysfunction hypothesis, bilateral lesions of the intralaminar nuclei of the thalamus, which is a collection of neurons including the centromedian-parafascicular (Cm-Pf) nuclei region, leads to reduction of tic symptoms in TS patients (Robertson et al., 1990). These and other lesioning studies demonstrated clinical improvement (Hassler and Dieckmann, 1970, Rickards et al., 2008) and the first reports of Cm-Pf high frequency TS DBS were later perfomed by Visser-Vandewalle and colleagues (Visser-Vandewalle et al., 2003). According to The International Tourette Deep Brain Stimulation Registry and Database, the most common brain target utilized worldwide has been the Cm-Pf region of thalamus (48.3% of all leads in the publicly available database) (Tourette Syndrome Association, 2019).

Current Cm-Pf targeting approaches for TS DBS are non-standardized and can result in substantial variability in final lead placement (Johnson et al., 2019). There is a paucity of literature focused on human microelectrode mapping in the Cm-Pf region as a potential technique to guide intraoperative DBS lead placement (Shields et al., 2008,, Warren et al., 2020). As approximately 46% of DBS failures are due to lead misplacement (Okun et al., 2005), techniques that may improve targeting accuracy and intraoperative confirmation are extremely desirable. For example, previously we observed a Cm-Pf region TS DBS case where there was no benefit from DBS treatment following 4 months of programming and stimulation adjustment. Post-surgical measurement of the lead location as well as the functional neurophysiological recordings drawn from monthly post-operative monthly visits indicated that the ventral portion of the lead was likely placed in the ventralis intermediate (Vim) nucleus instead of Cm-Pf region. Following subsequent lead revision surgery, the patient achieved better treatment outcomes (Cagle et al., 2020). This case highlights the importance of accurate lead placement and it should motivate continued efforts to refine neurosurgical targeting strategies.

Current targeting approaches of the Cm-Pf region typically involve an anterior-lateral entry angle mainly to avoid a trajectory through the lateral ventricles. This trajectory often proceeds through the Vim nucleus region immediately dorsal of the Cm-Pf region. The Vim nucleus is a well-studied motor nucleus of thalamus and common DBS target for essential tremor (Basha et al., 2014, Opri et al., 2019). In contrast, the Cm-Pf region has been investigated extensively for involvement in attention and limbic networks (Saalmann, 2014, Minamimoto and Kimura, 2002). Given the common use of a trajectory involving the Vim nucleus as well as the distinctive roles of both Cm-Pf region and Vim nucleus, we hypothesized that a functional mapping task could be utilized during awake DBS surgery. We posited that this task could differentiate the signal between the Vim and Cm-Pf and serve as a possible confirmatory marker of a lead placed in the intended Cm-Pf region.

## 2 Materials and methods

### 2.1 Study overview

This observational study was part of a larger IRB-approved (IRB#201300850, NCT02056873) clinical trial of female and male human patients with TS undergoing DBS treatment. All participants provided written informed consent. Participants were implanted with a sensing-enabled DBS device (Activa PC + S, Medtronic PLC, MN) for the primary purpose of identifying neural correlates of tics (Cagle et al., 2020) and for implementing closed-loop stimulation. As part of this clinical trial, participants returned to University of Florida (UF) for postoperative macroelectrodes local field potential (LFP) recordings and DBS programming occurred at monthly visits primarily during the first six months following DBS surgery.

In addition to completing the primary objectives of the parent clinical trial, a subset of these participants (N = 5) completed a modified, rewarding Go/No-Go task post-operatively at multiple visits while LFP signals were acquired. To confirm and replicate the results obtained from these participants, a separate subset of participants (N = 2) completed the task intra-operatively during Cm-Pf region DBS lead implantation. In this paper, we present the complete dataset from the Go/No-Go task performed in both the post-operative and intra-operative settings.

### 2.2 Study participants

The inclusion criteria included a DSM-V diagnosis of TS, Yale Global Tic Severity Scales (LECKMAN et al., 1989) > 35/50 for at least 12 months, and a motor tic subscore > 15. The tics must have been disabling to the patient, causing severe distress, possible self-injurious behavior, and/or quality of life disruption. We did not exclude patients with ADHD, OCD, or depression provided that the tics were the major issue prompting surgical intervention. Patients were also required to have failed trials of at least three dopamine blocking drugs and one trial of an alpha-2 adrenergic agonist prior to the DBS surgical intervention. Patients exhibiting additional unstable psychiatric disorders were excluded.

Across all seven participants, the average age was 33.66 ± 3.81 years (mean ± standard error) with a disease onset age of 9.0 ± 1.05 years. The postoperative data in this study are from participants consenting to testing after completing all other motor tasks required in the parent trial. Due to differences in recruitment timeframes, some participants completed more recording sessions than others and thus more bipolar contact pairs could be tested prior to exiting the study (see below; Table 1). The intraoperative patients were included in the same table for easier comparison of behavioral performance in the surgery room. The optimal stimulation contacts were provided for the time of recording.

**Table 1:** Overview of the post-operative recording sessions and behavioral data.

Participant ID	Session ID	Sensing Location (Left Hemisphere)*	Sensing Location (Right Hemisphere)*	Reaction Time (ms)	Accuracy (%)	Optimal Contact (L)	Optimal Contact (R)
Postop Subject #1	1	N/A	E00-E01	569.81	97.50	E01-CAN	E01-CAN
Postop Subject #2	1	E00-E01	N/A	520.50	100.00	E01-CAN	E01-CAN
Postop Subject #2	2	E00-E02	N/A	537.99	99.17	E01-CAN	E01-CAN
Postop Subject #2	3	E02-E03	N/A	533.04	99.17	E01-CAN	E01-CAN
Postop Subject #2	4	E01-E03	N/A	535.71	97.50	E01-CAN	E01-CAN
Postop Subject #3	1	E00-E01	E00-E01	581.13	97.50	E01-CAN	E01-CAN
Postop Subject #3	2	E00-E01	E00-E01	600.15	96.67	E01-CAN	E01-CAN
Postop Subject #3	3	E02-E03	E02-E03	572.70	97.50	E01-CAN	E01-CAN
Postop Subject #3	4	E02-E03	E02-E03	565.39	96.67	E01-CAN	E01-CAN
Postop Subject #4	1	E00-E01	E00-E01	579.60	99.17	E01-CAN	E01-CAN
Postop Subject #4	2	E00-E02	E00-E02	531.34	100.00	E01-CAN	E01-CAN
Postop Subject #4	3	E00-E01	E00-E01	616.49	99.17	E01-CAN	E01-CAN
Postop Subject #4	4	E01-E02	E01-E02	528.78	100.00	E01-CAN	E01-CAN
Postop Subject #4	5	E01-E03	E01-E03	509.85	98.33	E01-CAN	E01-CAN
Postop Subject #4	6	E02-E03	E02-E03	595.66	100.00	E01-CAN	E01-CAN
Postop Subject #5	1	N/A	E00-E01	662.56	95.83	E01-CAN	E01-CAN
Postop Subject #5	2	N/A	E00-E01	645.18	95.00	E01-CAN	E01-CAN
Postop Subject #5	3	N/A	E02-E03	649.10	96.67	E01-CAN	E01-CAN
Intraop Subject #1	1	All Contacts*	All Contacts	596.78	95.00	E02-CAN	E02-CAN
Intraop Subject #2	1	All Contacts	All Contacts	589.63	95.00	E01-E02	E02-CAN

* Contacts are numbered E0 (most ventral) to E3 (most dorsal).

### 2.3 Surgery

Participants underwent simultaneous bilateral electrode implantation of CM-Pf region DBS. A Cosman-Roberts-Wells headframe was placed and a stereotactic CT scan was obtained for co-registration to a preoperative MRI. The pre-operative MRI was acquired using a 3T SIEMENS MRI scanner (Siemens Medical Solutions, PA) per standard surgical procedures for anatomical targeting (Maling et al., 2012, Sudhyadhom et al., 2009). A Schaltenbrand-Bailey deformable atlas was manually fitted on each participant’s pre-operative MRI using a 9 degrees-of-freedom affine transformation in the UF-designed targeting software. Trajectories that were selected traversed the dorsal-medial aspect of the Vim nucleus en-route to the Cm-Pf region (Fig. 2). Microelectrode targeting and general anesthesia were not performed. Model 3387 DBS electrodes (Medtronic PLC, MN) were implanted at the targeted locations. Ground and reference electrodes were placed on the scalp for intraoperative macroelectrode LFP recordings. At this point the two intraoperative participants described in this study then completed the Go/No-Go task.

### 2.4 Go/No-Go Task

We hypothesized that the Cm-Pf nuclei of thalamus will be activated by tasks that are involved in the attention of the participants, which are known to elicit responses in animal models (Minamimoto and Kimura, 2002). The modified Go/No-Go task is a complicated task that requires attention of the participants in order to achieve high accuracy. Although there are multiple segments of the task worthy of investigation in the traditional context of Go/No-Go task such as responses to reward, they are beyond the primary goal of the study, which was to use visual attention physiology for targeting the Cm-Pf nuclei region of thalamus. The visual cue phase is the most attention-demanding segment of the task, in which the participants pay attention to the type of stimuli presented and attempt to react accurately. The full Go/No-Go task will be presented in this section, however only the signals evoked by visual cue presentation, regardless of the Go or No-Go cue were considered for the main purpose of the study.

**Fig. 1:**
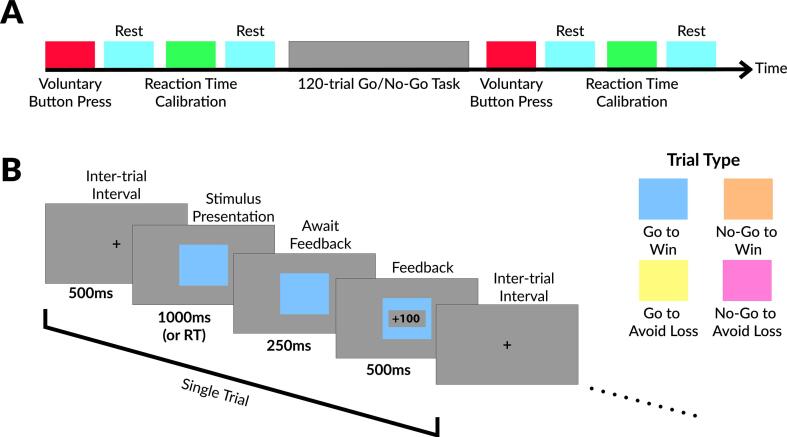
Task overview. A) Overall block design of the experiment. The experiment is divided into 3 sections: pre-task baseline, task, and post-task baseline. Each voluntary button press section is around 10 s based on once per second rate of pressing. Each resting period is 20 s, however, segments of motor activity as identified by video and EMG recording were discarded. The reaction time calibration was used to ensure the participants are able to react within the 1000 ms window. B) The task design and timing. Each trial includes 500 ms inter-trial interval, 1000 ms stimulus presentation, 250 ms wait, and 500 ms feedback. The stimulus presentation timing will be shortened if reaction occurred early. The 4 possible colors (or trial type) are displayed under the task overview.

The experiment consists of four 20-second baseline recordings (two before the task and two after the task), two self-paced 10-button pressing recordings (one before the task, and one after the task; data not shown), two reaction time tests (one before the task, and one after the task; data not shown), and a Go/No-Go task. The task portion consisted of 120 trials for postoperative recordings and shortened version of 60 trials for intraoperative recordings to reduce time taken in the operating room. The task setup is outlined in Fig. 1. The participant was presented with a colored rectangle (visual stimulus) with four possible colors (blue, orange, yellow, purple), each corresponding to a unique condition: 1) press to receive an award (blue; Go To Win), 2) do not press to receive an award (orange; No Go To Win), 3) press to avoid losing (yellow; Go To Avoid Loss), 4) do not press to avoid losing (purple; No Go To Avoid Loss). The order of stimuli was random. The stimuli were presented for 1000 ms followed by a 250 ms wait period after participants reacted (by pressing button or not pressing button) to the stimuli (Fig. 1). Based on the participant’s reaction, feedback was then displayed for 500 ms. Win outcomes were +100 points, lose outcomes were −100 points, and avoid-loss outcomes were +0 points. After feedback a cross was displayed on the screen for 500 ms during the inter-trial interval as the pre-trial baseline. The feedback portion of the task was displayed mainly to provide motivational encouragement to complete the task. A pressure-based push button was given to the participants to hold in their dominant hand for responding during the task. The sensor was connected to the external synchronization box (Alcantara et al., 2020), which was connected to the external amplifier’s digital input (see below).

**Fig. 2:**
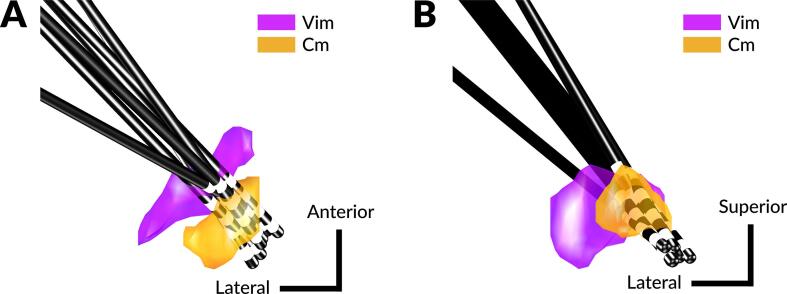
Post-operative study participants electrode placement overview. The atlas overlay was based on the Schaltenbrand-Baily atlas, and only the left atlas was displayed. All electrodes from right hemisphere were moved to left hemisphere space by inverting the Y-axis (laterality) values. A) Electrodes shown in the axial view. B) Electrodes shown in the coronal view. (Vim: ventral intermediate nucleus; Cm: centromedian nucleus.)

### 2.5 Experimental setup

The Go/No-Go task was designed and written in BCI2000 (Schalk et al., 2004). Therefore, all state triggers, markers, and behavioral data were collected in the same framework and was set at a 2400 Hz sampling rate. An external monitor with a resolution of 1280 × 1024 was placed in front of the patient with an appropriate viewing angle. Participants were instructed how to play the game beforehand, provided an opportunity to practice, and all 4 possible colors were shown to the patient in random order prior to each recording to ensure that the participants were able to differentiate all stimuli.

In the postoperative setting, all neural data were collected using an Activa PC + S system (Connolly et al., 2015), which is limited in recording capability to one bipolar channel per thalamic electrode. All neural recordings were collected using a 422 Hz sampling rate with a gain of 2000. A different bipolar contact pair was selected during each recording session for each patient. (Table 1). The bipolar contact pairs were chosen based on the best signal-to-noise ratio during motor tasks (data not shown) and in an effort to obtain recordings with maximum spatial differences spanning the electrode (dorsal vs ventral contacts). The dorsal and ventral electrode contacts refer to the recording contacts along the trajectory of the Medtronic 3387 deep electrode (Contact E00-E01 are more ventral and E02-E03 are more dorsal). The ventral electrode contacts were generally located within the Cm-Pf nuclei of thalamus while the dorsal electrode contacts were generally closer toward the VIM nucleus. The positive contact in the bipolar contact pairs was always the more ventral contact. LFP data were aligned with behavioral data using an external electromyography (EMG) system. At the beginning of the recording, two wireless EMG sensors were placed over the participant’s neck (where the electrode wires passed below), and a 5 Hz electrical stimulation was initiated for 3 to 10 s. In addition, the BCI2000 system delivered state triggers to an external synchronization box which then converted the signal to the EMG system for alignment with the BCI2000 data. Based on this setup, for both postoperative and intraoperative recordings of the Go/No-Go task, all neural data, EMG data, and game states were aligned for unified analysis. Data alignment was completed in MATLAB 2016a (Mathworks, Natick, USA), and the aligned BCI2000 data, EMG data, and neural data were stored as MAT-file (version 7.3).

In the intraoperative setting, DBS electrodes were connected to a g.tec HiAmp (Guger Technologies, Schiedlberg, Austria) external amplifier. Two separate corkscrew electrodes were placed in the scalp as the reference and grounding electrodes. The monopolar channels obtained during intraoperative sessions from an external amplifier were converted to bipolar recordings – always the more ventral contact minus the more dorsal contact – to resemble postoperative signals through post-processing. However, one recording error was made during the first intra-operative recording due to a mistake in the reference electrode selection. The reference for the first patient was the left hemisphere thalamic electrode contact 3 instead of the scalp corkscrew electrode, which led to different bipolar pairs used when comparing to other intraoperative leads (see below).

### 2.6 Imaging and lead measurements

Pre-operative T1-MRI and post-operative Stealth CT were obtained for each participant for lead measurements in anatomical space. The post-operative CT was acquired one month after surgery, and the post-operative CT was fitted to the pre-operative MRI using MATLAB multimodal co-registration for geometric transformation estimation (Image Processing Toolbox, MathWorks Inc, Natick, MA). After co-registration, the electrode position was measured in the T1-MRI space and reverse transformed into the Schaltenbrand-Bailey common atlas space for group analysis based on each participant’s individual surgical atlas transformation, that was manually fitted by the surgeon. The cartesian coordinate of a bipolar recording was expressed as the root-mean-square distance between the midpoint between the two contacts contributing to the bipolar recording and the target coordinate, which is the ventral Cm border (X: ±9.23, Y: −8.56, Z: 2.49) in the digitized Schaltenbrand-Bailey atlas space. All thalamic electrodes from the right hemisphere were moved to the left by negating the X-axis in the Schaltenbrand-Bailey atlas space as the deformable Schaltenbrand-Bailey atlas space is symmetric and each side of the atlas is fitted independently of the other.

### 2.7 Data analysis

The data analysis was performed in MATLAB 2016a (Math works Inc, Natick, MA). Behavioral results including reaction time (RT) and accuracy for each recording session were calculated to ensure that participants were engaged in the task. The accuracy was calculated as the number of correct responses (pressing for Go trials, and not pressing for No-Go trials) divided by the total number of trials. RT was calculated as the time from stimulus presentation to button press for the Go trials performed correctly.

The neural recordings were filtered between 1 and 30 Hz using a 3rd-order Butterworth filter with zero-phase digital filtering. Due to the recordings spanning multiple months, all neural data were normalized prior to group analysis. For each recording session, the neural data were z-score normalized based on the average signal during the four 20-second baseline recordings (Fig. 1). However, baseline recordings that contained extensive EMG activities were excluded from z-score calculation. This occurred in one of the baseline recordings in one subject only.

Since the primary signal of interest was the event-related potential (ERP) after stimulus presentation, the task recording was converted into 1000 ms epochs (-200 ms to 800 ms) around each visual stimulus presentation. Trials with incorrect responses or with overall amplitude more than 3 standard deviations above or below the mean amplitude at any time point were removed from the analysis to avoid artifacts from influencing the results. The remaining trials were averaged within each recording session to create the stimulus onset ERP for each session. Then, the ERPs from all recording sessions were averaged again to obtain a grand average ERP. The grand average ERP after stimuli presentation and before average reaction time was tested against 0 using Wilcoxon’s signed rank test. The most significant features were identified as the positive or negative deflections above or below 0 occurring for more than 50 ms at the group level. These features were extracted from each participant’s recording at the individual level by finding the maximum or minimum point within the feature window of each individual run for the positive and negative deflection, respectively.

To assess whether the ERP reflected anatomical specificity of the Cm-Pf, these feature magnitudes were used for a correlation with the z-position of the bipolar recording. Shapiro-Wilk test was performed on both the z-positions and the ERP features to determine whether parametric (i.e., Pearson) or non-parametric (i.e., Spearman) correlation should be used.

For the two intraoperative recordings, the neural signals were filtered and processed similar to the postoperative recordings. All recordings were manually converted to bipolar recordings in MATLAB by taking the difference of two adjacent contacts (i.e., E00-E01, E01-E02, and E02-E03), with the exception of intraoperative subject #1 left hemisphere, where a monopolar recording was used due to contact E03 inadvertently being selected as recording reference. ERPs for each bipolar recording were computed by averaging all trials, and both the positive peak feature and negative peak feature were extracted from the recordings. One-tail Wilcoxon rank sum test was performed between the three bipolar recordings based on a-priori hypothesis derived from postoperative recordings, namely, that the ventral contact pairs should have a stronger positive peak feature and a stronger negative peak feature compared to dorsal contact pairs.

## 3 Results

### 3.1 Study participants and behavioral results

All bipolar contact pairs used in the sessions, as well as the behavioral performance during each session, are provided in Table 1. A total of 18 sessions from five participants were recorded. There were no trials which were rejected due to large spikes in signals in postoperative patients. However, the intraoperative recordings contain more external electrical artifacts leading to large spikes manifesting with an amplitude that was 3 standard deviations above baseline. The percent of trials retained after rejection was 61.7% and 95%. The average reaction time for all sessions was 574 ms. Of the five post-operative participants, two of them had a unilateral neurostimulator with a depleted battery (Subject#1 left device and Subject#2 right device), and one participant had a malfunctioned sensing module in the implanted neurostimulator which prevented data collection (Subject #5 left device). Among the 18 sessions, 10 sessions were simultaneous bilateral recordings. To simplify the analyses, simultaneous bilateral recording was treated as 2 independent recordings for thalamic electrodes in the left and right hemisphere, which led to a total of 7 electrodes across 5 participants. The positions of all electrodes are shown in Fig. 2.

### 3.2 Visual evoked potential features

**Fig. 3:**
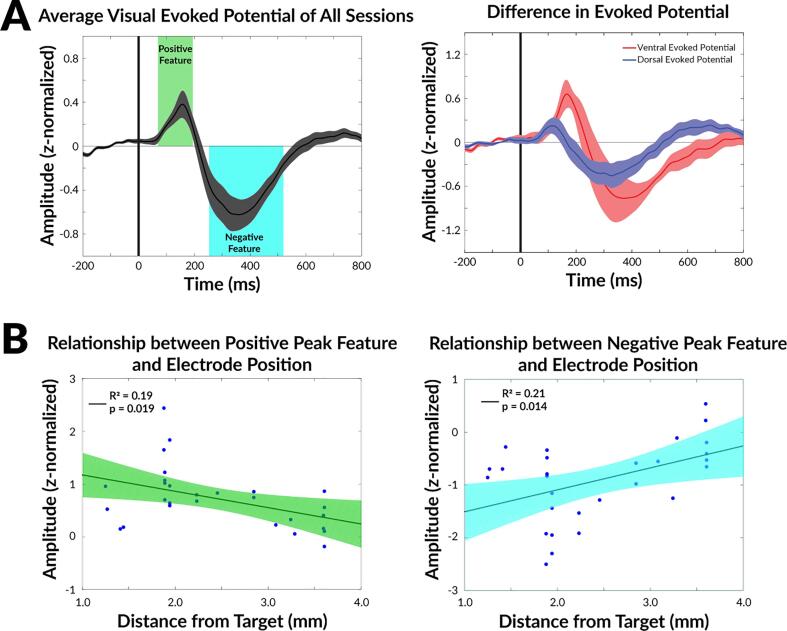
Summary of the post-operative visual evoked potential features. A) Two features, a positive feature and a negative feature, were identified in the grand average ERP, the average neural response from all patients across all recording sessions of different electrode contact pairs. The positive feature occurred between 75 ms and 192 ms. The second feature was a negative deflection occurring between 256 ms and 526 ms. The dark gray interval represents 1 standard error above and below the grand average ERP. B) The average ERP of recordings from the recording contacts more dorsal in the electrode and recording contacts more ventral in the electrode were showed. The dark red and dark blue interval represents 1 standard error above and below the average ERPs. C) Correlation of the maximum peak during positive feature period and D) minimum peak during negative feature period with the electrode position (measured as mm above AC-PC line). Both features are statistically correlated with the electrode position, with the positive peak feature emerging as the stronger feature. Colored shaded regions indicate the 95% confidence interval of the linear fit. (AC-PC: anterior commissure-posterior commissure; ms = milliseconds; mm = millimeters). (For interpretation of the references to colour in this figure legend, the reader is referred to the web version of this article.)

The grand average ERP of all recording sessions from all patients, which contain recordings from both the ventral electrode contact pairs and dorsal electrode contact pairs, were computed and shown in Fig. 3A. Two deflections above or below 0 were identified. The first was a positive deflection occurring between 75 ms and 192 ms after visual stimuli presentation with a peak occurring at 160 ms. The second feature was a negative deflection occurring between 256 ms and 526 ms with a peak occurring at 360 ms. The average ERP from the ventral and dorsal recordings of the electrode are presented in Fig. 3B. The dorsal recordings (Blue) show weaker evoked potentials when compared to the ventral recordings (Red).

Shapiro-Wilk test indicated that neither the electrode positions nor the amplitude features were normally distributed, thus Spearman’s correlations were used for the positive and negative peak features. The correlations of feature strength and position in the Z axis are shown in Fig. 3B. Both features correlated significantly with the recording locations. Namely, deeper electrodes were associated with more positive (p = 0.019) and more negative (p = 0.014) features.

### 3.3 Intra-operative verification

**Fig. 4:**
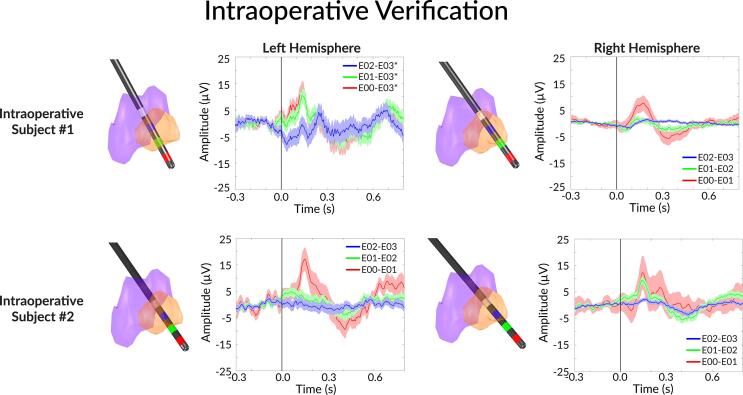
Examples of the two of the intra-operative study participants’ electrode positions and their respective visual evoked potentials. Top row are results from intraoperative participant #1. Bottom row are results from the intraoperative participant #2. The left electrode positions and evoked responses are derived from the left hemisphere, and right electrode positions and evoked responses are derived from the right hemisphere. The right electrode position is mirrored to the left hemisphere to be fit in the same atlas. E00 to E03 contacts are numbered from the most distal contact to the most proximal contact in the Medtronic 3387 electrode. The shaded areas are +/− 1 standard error from the mean evoked response.

Electrode positions and LFP signals from the two intraoperative participants are provided in Fig. 4. All electrodes revealed a stronger a positive peak around ventral contact pairs, which tended to be just ventromedial to the CM region in the Schaltenbrand-Bailey atlas (Fig. 4). No significant peaks were found in VIM regions.

Wilcoxon rank sum test for the positive feature in intraoperative subject #1 showed that the left hemisphere E00-E03 and E01-E03 contact pairs were not statistically different from E01-E03, but E00-E03 is higher than E02-E03 (p = 0.0257). In the right hemisphere, E00-E01 had a stronger peak feature than both E01-E02 and E02-E03 (p < 0.0458 and p < 0.0222, respectively), but E01-E02 and E02-E03 were not different from each other. There were no statistical differences between the negative peak feature from all three bipolar pairs in the left hemisphere. For the right hemisphere, E00-E01 and E01-E02 had stronger negative peak features than E02-E03 (p < 0.0321 and p < 0.0028, respectively).

Wilcoxon rank sum test for the intraoperative subject #2 positive feature showed that left hemisphere E00-E01 was significantly higher than E01-E02 and E02-E03 (p < 0.0070 and p < 0.0013, respectively), but E01-E02 and E02-E03 were not different from each other. In the right hemisphere, E00-E01 was not statistically different from E01-E02 and E02-E03, due to high variability, but E01-E02 was significantly higher than E02-E03 (p < 0.0001). There were no statistical differences between the negative peak feature from all three bipolar pairs in the left hemisphere. For the right hemisphere, E00-E01 had stronger negative peak features than E01-E02 and E02-E03 (p < 0.0344 and p < 0.0115, respectively). In summary, the signals from both patients revealed the strongest response in the ventral contacts of the electrode which were closer to (CM-Pf region and immediately inferior to CM) and weaker in strength in recording location closer to the VIM region.

## 4 Discussion

Using an attention-driven cognitive task, in this study we found distinct LFP activity patterns within different regions of the thalamus in TS patients undergoing Cm-Pf DBS. ERP features revealed a greater strength when recorded closer to the Cm-Pf region along the planned DBS trajectory when compared directly to features from recording closer to Vim region. Interestingly, we identified two significant ERP features occurring after visual stimuli presentation: an early positive peak within the first 200 ms of visual stimuli presentation, and a negative peak shortly after. Both features’ strengths were significantly correlated with the electrode position. In contrast to the Cm-Pf nuclei region, bipolar contact pairs closer to the Vim region showed little to no ERP response following visual stimuli. This result was observed in all 5 post-operative recording participants and was robustly present across multiple sessions.

To confirm these results in the intra-operative setting, we performed the same task during DBS implantation surgery for two additional participants. Although the referencing error in intra-operative subject #1 prevented side-by-side bipolar contact pairs for smaller LFP volumes, the visual evoked potential was present and display greater strength in the more ventral area along the DBS lead. Both positive features and negative features existed, but the positive feature was more prominent and seems to exist even in a monopolar configuration (intraoperative subject #1, left hemisphere). Intra-operative physiology for the Cm-Pf region targeting has been explored in limited studies. Warren et al. presented Cm targeting for DBS in 19 epilepsy patients. They observed reduced firing rates as the microelectrode trajectory entered the Cm region from the ventrolateral nucleus, but this group level result was not confirmed in 20–25% of the patients in their cohort (Warren et al., 2020). Shields et al. presented a case of Cm-Pf nuclei targeting using microelectrodes, however, the main differentiating strategy was based on thalamic border identification but not differentiating nuclei within thalamus (Shields et al., 2008).

Our potential solution was a novel LFP-based functional approach using the modified Go/NoGo task. This approach was intuitive because the Cm-Pf nuclei has been well described to be involved in attention. Most of the early evidence for the function of CM has been drawn from non-human primates and rodent studies involving ablation or visual cue pressing tasks (Minamimoto and Kimura, 2002, Kato et al., 2011). Recent human studies of the Cm-Pf nuclei also provides additional support to the role of attention processing during oddball tasks (Raeva, 2006, Schepers et al., 2017, Beck et al., 2020). In contrast, the Vim nucleus of the thalamus is heavily involved in motor control (Opri et al., 2019, Sommer, 2003). With an anterior-lateral entry angle, the DBS electrode typically passes through Vim nucleus before entering Cm-Pf nuclei of thalamus, and our results support and leverage the distinct functions of these nuclei regions. However, we were not able to examine the signals from various other nuclei within thalamus because they were not part of the surgical trajectory for these patients.

One limitation of all LFP macroelectrode recordings is that the specific anatomical source of the signals remains unknown. Originally, we selected an engaging task to elicit an attention-based signal from the Cm-Pf region of thalamus. However, combining post-operative imaging and multiple sessions with different electrode contact pairs, we observed that the strongest signal appears to originate a few millimeters inferior/posterior to the Cm nucleus. This can be seen clearly in intra-operative recordings from subject #1 left electrode (Fig. 4A), which is more inferior than the others, and from intra-operative subject #2′s right electrode (Fig. 4D), which is more posterior than the others. Both (subject #1 and #2) electrodes revealed a similar strength of the visual evoked potential in the ventral contact pairs, while other electrodes showed a gradient of a weak to strong potential as the recording configuration spanned proximally to distally.

It is likely that if the electrodes are implanted too deep along the intended trajectory, we may possibly measure activity from the pulvinar nucleus region of thalamus. The pulvinar nucleus of thalamus, similar to the Cm-Pf nuclei, is heavily involved in cognitive processing, especially higher-order visual processing (Snow et al., 2009, Fischer and Whitney, 2012 Sep 11, Kaas and Lyon, 2007). Positioned just millimeters posterior to Cm-Pf nuclei, the pulvinar nucleus could therefore potentially influence the LFP recorded from the distal contact pairs drawn from the DBS electrodes placed more posteriorly. The study was IRB approved and carried out in a manner to limit any additional risks to the study participants; therefore, electrophysiology signals were recorded based on the neurosurgeons’ decision on the region of placement. We were not ethically able to record beyond the intended targeting position in a deliberate effort to prevent additional tissue damage in thalamus. Our strategy of targeting the Cm-Pf nuclei region of thalamus should be based on a *the emergence of evoked potentials in the ventral recording contacts but weak to absence in dorsal recording contacts* along the DBS electrode as opposed to simple identification of the strongest response to the visual stimuli.

In addition, it is unclear if the recording region is influenced by proximity to the visual pathways giving rise to evoked potentials or due to changes in the visual field itself. The current study design was not able to eliminate the possible source of the evoked potential from the visual change, however, further study should incorporate the visual oddball design to control for visual changes while focusing on the attentional aspects of the oddball stimuli.

This study had several important limitations. First, this was a pilot study and included different numbers of recordings from a small group of patients. However, despite the recording limitation, data were normalized, and we demonstrated the evoked potentials can be identified in individual level in the operating room setting. Second, we did not perform a real-time mapping along the full trajectory of the DBS electrode. The current approach was designed to measure the presence of evoked potential, which is shown to correlate with the proximity to the CM-Pf nuclei region in Fig. 3, after electrode was placed at the intended target location based on anatomical targeting approach. Although this task takes less than ten minutes to complete, we envision an even simpler task with less involvement of the patient during DBS electrode insertion would be a more practical way to translate the method into common practice. In addition, future studies can incorporate the clinical outcomes of each patient and their optimal therapeutic stimulation contacts to verify that the evoked potential can serve as important biomarker for optimal therapy locations.

Overall, we have presented a novel functional mapping approach for the Cm-Pf nuclei region of thalamus which can be used for TS DBS awake human neurosurgery. The method may also be applied to CM region targeting of other neurological and neuropsychiatric disorders beyond TS, however this method would require confirmation that the findings from our study are not unique to the TS population. Future work should confirm these results prospectively and in a larger sample size. We envision that the task could be further refined, and auditory testing could be explored as a way to avoid pulvinar influences on the visual responses.

## Funding

This work was supported by R01NS096008, the Norman Fixel Institute for Neurological Diseases, the University of Florida Pruitt Family Endowed Faculty Fellowship, National Science Foundation PECASE 155348, and 10.13039/100000002National Institutes of Health/National Center for Advancing Translational Sciences Clinical and Translational Science Awards UL1TR001427, KL2TR001429, and TL1TR001428 to the 10.13039/100007698University of Florida. This work was also supported by National Institutes of Health National Institute of Neurological Disorders and Stroke Award F30NS111841.

## CRediT authorship contribution statement

**Jackson N. Cagle:** Conceptualization, Methodology, Software, Investigation, Writing - original draft. **Robert S. Eisinger:** Conceptualization, Methodology, Software, Validation, Writing - original draft. **Marshall T. Holland:** Writing - review & editing. **Kelly D. Foote:** Resources, Supervision. **Michael S. Okun:** Resources, Supervision, Writing - review & editing, Writing - review & editing. **Aysegul Gunduz:** Resources, Supervision, Writing - review & editing.

## Declaration of Competing Interest

The authors declare that they have no known competing financial interests or personal relationships that could have appeared to influence the work reported in this paper.
